# Prevalence of risk behaviors and correlates of SARS-CoV-2 positivity among in-school contacts of confirmed cases in a Georgia school district in the pre-vaccine era, December 2020–January 2021

**DOI:** 10.1186/s12889-021-12347-7

**Published:** 2022-01-14

**Authors:** Marisa Hast, Megan Swanson, Colleen Scott, Emeka Oraka, Catherine Espinosa, Eleanor Burnett, Esther A. Kukielka, Marion E. Rice, Lemlem Mehari, Jazmyn McCloud, Danielle Miller, Rachel Franklin, Jacqueline E. Tate, Hannah L. Kirking, Elana Morris

**Affiliations:** 1 CDC COVID-19 Response, Centers for Disease Control and Prevention, 1600 Clifton Rd. NE, 30329 Atlanta, GA USA; 2 General Dynamics Information Technology, 3150 Fairview Park Dr, Falls Church, VA 22042 USA; 3 Epidemic Intelligence Service, Centers for Disease Control and Prevention, 1600 Clifton Rd. NE, 30329 Atlanta, GA USA; 4 Cobb & Douglas Public Health, 1650 County Services Pkwy, 30008 Marietta, GA USA; 5 Georgia Public Health Laboratory, 1749 Clairmont Rd, 30033 Decatur, GA USA

**Keywords:** SARS-CoV-2, COVID-19, Schools, Students, Children, Behaviors, Behavioral epidemiology

## Abstract

**Background:**

There is a continuing risk for COVID-19 transmission in school settings while transmission is ongoing in the community, particularly among unvaccinated populations. To ensure that schools continue to operate safely and to inform implementation of prevention strategies, it is imperative to gain better understanding of the risk behaviors of staff and students. This secondary analysis describes the prevalence of COVID-19 risk behaviors in an exposed population of students and school staff in the pre-vaccine era and identifies associations between these behaviors and testing positive for SARS-CoV-2.

**Methods:**

From December 2020–January 2021, school staff and students exposed to confirmed COVID-19 cases in a Georgia school district were tested for SARS-CoV-2 and surveyed regarding risk behaviors in and out of school. Prevalence of risk behaviors was described by age group and school level, and associations with SARS-CoV-2 positivity were identified using chi squared tests.

**Results:**

Overall, 717 students and 79 school staff participated in the investigation; SARS-CoV-2 positivity was 9.2%. In the 2 weeks prior to COVID-19 exposure, 24% of participants reported unmasked indoor time at school, 40% attended social gatherings with non-household members, and 71% visited out-of-school indoor locations, including 19% who ate indoors in restaurants. Frequencies of risk behaviors increased by age. Among students, 17% participated in school sports, of whom 86% participated without a mask. SARS-CoV-2 positivity was significantly associated with school sports and unmasked time in sports. Among K-5 students, positivity was associated with exposure to a teacher index case.

**Conclusions:**

This analysis highlights the high prevalence of risk behaviors in an unvaccinated population exposed to COVID-19 in school and identifies an association between student sports participation and SARS-CoV-2 positivity. These findings illustrate the importance of school-level prevention measures to reduce SARS-CoV-2 transmission, including limiting close-contact indoor sports and promoting consistent mask use in unvaccinated individuals. Future research could explore the role of community vaccination programs as a strategy to reduce COVID-19 transmission and introductions into school settings.

**Supplementary Information:**

The online version contains supplementary material available at 10.1186/s12889-021-12347-7.

## Background

In March 2020, the COVID-19 pandemic caused widespread closures of schools throughout the U.S., impacting up to 55 million students in 124,000 schools [1, 2]. Studies suggested that these closures may have negatively impacted student learning and the mental health of children and families, [3–5] and due to the importance of in-person education for children, families, and communities, many schools reopened for face-to-face instruction in the 2020/2021 academic year [6, 7]. However, due to their potential for high population density and frequency of close contact between students or staff, risks for introductions and transmission within the school setting persist while transmission of SARS-CoV-2 is ongoing in the community, particularly in populations where vaccination remains low [7, 8]. Instances of widespread secondary transmission of SARS-CoV-2 have been documented in school settings when comprehensive prevention strategies are not implemented [9]. To ensure that schools remain open, can continue to operate as safely as possible during the COVID-19 pandemic, and to inform implementation of prevention strategies, better understanding of the risk behaviors of staff and students relevant to in-school transmission is imperative.

Prevention strategies such as mask use and physical distancing have been identified as significant protective factors for reducing SARS-CoV-2 transmission in schools, while risk factors have included close-contact indoor sports, eating meals in close proximity, and attending social gatherings with persons outside the household [10–14]. Similarly, the effectiveness of prevention strategies in the community are well documented, while activities such as dining in restaurants, attending church, and group fitness classes have been identified as risk factors for transmission [15–25]. However, information on the prevalence of risk behaviors among school populations and how these behaviors impact risk of transmission in educational settings is sparse, and few studies have formally assessed the direct relationship between individual behaviors and SARS-CoV-2 transmission in schools. Understanding the extent to which students and school staff engage in risk behaviors may contribute to developing policies and practices that could help to reduce introductions and transmission of SARS-CoV-2 in school settings in order to limit school closures and maximize opportunities for in-person learning.

To describe the extent of transmission of SARS-CoV-2 in schools, the Centers for Disease Control and Prevention (CDC) partnered with the Georgia Department of Health and Cobb & Douglas Public Health to investigate transmission in a Georgia public school district. Results of this investigation showed that teacher-to-teacher and teacher-to-student transmission were a central element of transmission chains in this district [26, 27]. To further inform school and community efforts to reduce SARS-CoV-2 transmission in schools, this secondary analysis utilized data collected during the school transmission investigation to describe the prevalence of risk behaviors relevant for SARS-CoV-2 transmission in this population of unvaccinated students and school staff and to identify associations between these behaviors and testing positive for SARS-CoV-2 in the era prior to widespread vaccination availability.

## Methods

### Population and Setting

The population under investigation included all 12 public schools in a school district located in the Atlanta metropolitan area in the US. The school district includes approximately 1,400 staff and 8,500 students across one high school (grades 9-12), two middle schools (grades 6-8), eight elementary schools (grades K-5), and one early learning center (pre-K). The period of investigation was December 1, 2020–January 26, 2021, including a 10-day holiday break. Seven-day county incidence of COVID-19 was peaking at this time, with a high of 705 cases per 100,000 on January 13, 2021 [27]. By the end of the investigation period, educational staff and children were still not eligible for COVID-19 vaccination in Georgia, and the state vaccination rate was only 2.6% [28].

During the investigation period, parents or guardians chose whether their children went to school fully online or fully in-person four days per week, with all students attending online on Fridays. All students had the option to participate in school sports in person. In accordance with public health recommendations, [7] the district used a number of measures to reduce the risk of transmission in schools, which are described in detail elsewhere [27]. In brief, these included mandatory mask use indoors and on the school bus, enhanced hand hygiene, three-sided plastic dividers on student desks, and spacing desks further apart. There were exceptions to mandatory mask use during meals, mask breaks (times students could remove their masks for short periods), outdoor recess, and indoor and outdoor physical activity.

### Investigation Procedures

During the study period, school staff or students in the school district with confirmed SARS-CoV-2 infection were identified by the health department or self-report and are included here as index cases. School district staff conducted contact tracing to identify all persons who were exposed to an index case in a school setting, defined as being within six feet for a cumulative ≥15 minutes within 24 hours during the infectious period (starting 48 hours prior to the case’s positive test if asymptomatic, or 48 hours prior to symptom onset) [29]. These individuals are referred to as contacts. Methods for contact tracing included interviews with index cases and review of classroom and bus seating charts. This process helped determine characteristics of exposure, including location (e.g., classroom, bus), school role of the index case, and whether the contact was exposed to multiple index cases concurrently [27]. The school district then reported the contact and their characteristics of exposure to the CDC investigation team.

The CDC team called each contact/their guardian to explain the investigation, request verbal informed consent, and offer the contact COVID-19 testing at no cost. To collect additional information on the contact and the context of their in-school exposure, a brief survey was administered over the phone in English, Spanish, or Portuguese. The survey was developed for the purposes of this study in consultation with subject matter experts and included questions on demographics, behaviors in school that might modify their exposure level (e.g., mask use), and a variety of behaviors in and out of school that have previously been identified as high-risk (e.g., dining in restaurants) [10–25]. All behaviors were collected for the two weeks prior to the exposure event in order to best capture any factors that could inform risk in the epidemiologically relevant time period. The survey tool can be found in supplementary materials. Questions were asked directly to the student if they were in high school and the parent consented, otherwise they were asked to the parent with the student present. Responses were entered into Epi Info version 7.2.3.1 (CDC, Atlanta, GA), and testing date was recorded in a call log. If the investigation team was unable to reach the contact, they attempted to call back at least twice.

Drive-through COVID-19 testing was provided two hours per day during December 4–24, 2020, and January 8–26, 2021, for a total of 40 testing days. Appointments for testing were preferentially scheduled five to seven days post-exposure per standard guidelines but could be scheduled up to ten days from last exposure if necessary [30]. At the testing site, adult contacts or guardians provided written informed consent, and the investigation team collected anterior nasal swabs. Samples were placed in a cooler and transported to the Georgia Public Health Laboratory for analysis within three hours of collection. This activity was reviewed by CDC IRB, and was approved to be conducted as a public health activity consistent with applicable federal law and CDC policy, e.g. 45 C.F.R. part 46, 21 C.F.R. part 56; 42 U.S.C. §241(d); 5 U.S.C. §552a; 44 U.S.C. §3501 et seq.

### Laboratory Methods

All samples were refrigerated at 2-8°C and were tested within 24 hours of receipt at the Georgia Public Health Laboratory. Viral nucleic acid material was extracted using a PerkinElmer Chemagic 360 platform (PerkinElmer, Waltham, MA). Real-time reverse transcriptase polymerase chain reaction (RT-PCR) testing was conducted using the PerkinElmer New Coronavirus Nucleic Acid Detection Kit on a 7500 Fast Dx Real-Time PCR system (Applied Biosystems, Foster City, CA) for qualitative detection of SARS-CoV-2 nucleic acid. All contacts received RT-PCR results by email within 48 hours of sample collection.

### Data Management

**Fig. 1:**
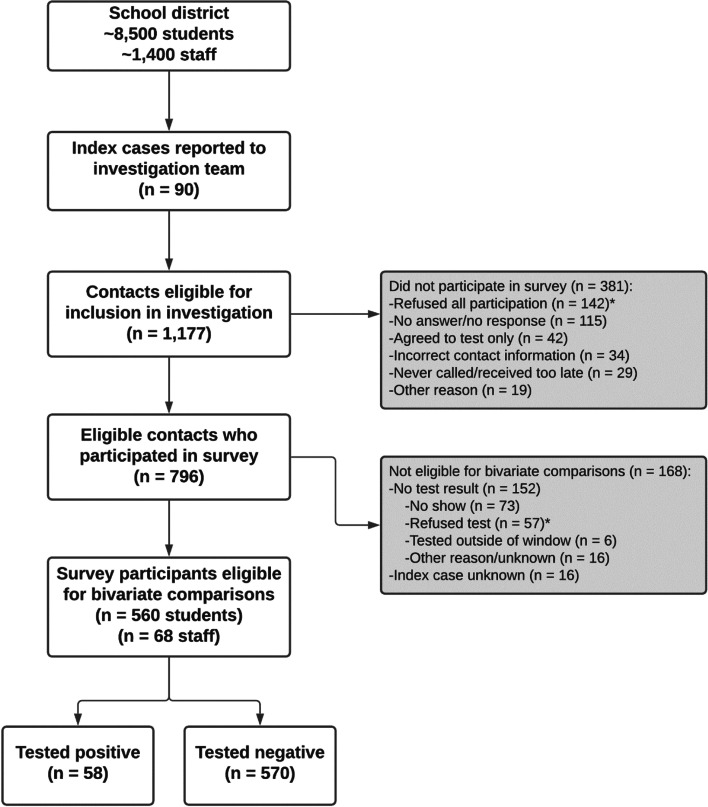
Flowchart of participation among staff and students participating in an investigation on in-school transmission in a school district in Georgia, December 2020–January 2021. Participants were eligible for bivariate comparisons if they had a SARS-CoV-2 test result and the characteristics of their index case were known. Reasons for non-participation marked with an asterisk indicate participant refusal of survey and/or test

Data from call logs, RT-PCR testing, and participant surveys were merged into a master dataset. Participants were included in this analysis if they completed the survey and had complete information on their staff vs. student role and school level (Fig. 1). For analyses involving SARS-CoV-2 positivity, participants were retained if they also had a recorded COVID-19 test from CDC testing or another source within 5-10 days of their in-school exposure. If a previously negative participant had an independent exposure in school more than two weeks after their first exposure and completed an additional survey and test, that participant was included in the dataset separately for each exposure. In the rare instance that a participant was exposed to multiple index cases concurrently, this was counted as a single exposure event in the analysis, but the number of index cases was recorded.

### Statistical Analyses

Data were analyzed using SAS 9.4 (SAS Institute, Cary, NC) and R 3.6.1 statistical software (The R Foundation). All analyses were stratified by school role and school level, with the three categories defined as staff, elementary school students (grades pre-K–5), and middle/high school students (grades 6–8 and 9–12). Frequencies are presented for demographics, school-reported exposure characteristics (e.g., exposure setting, staff vs. student index case role), and self-reported risk behaviors in and out of school in the two weeks before exposure (e.g., mask use, participation in school sports, visits to non-school indoor locations). Middle/high school students may have reported sports participation as either formal inter-school sports leagues or informal in-school athletics, while elementary school students had only informal in-school athletics. Staff reported participation in sports as a coach or sponsor. For staff, frequencies also are presented for in-school interactions with other staff, including in-person meetings, indoor lunch, other social time, and unmasked time with other staff.

For students, bivariate comparisons were conducted by school level using chi-squared or Fisher’s exact tests to identify associations between SARS-CoV-2 positivity, demographic factors, characteristics of exposure, and self-reported behaviors. Small sample size precluded bivariate comparisons for staff participants, so frequencies were calculated by SARS-CoV-2 test result and are presented in supplementary materials. Multivariate logistic regression was attempted among all variables significant at the *P* = 0.1 level using stepwise regression and Akaike information criterion optimization methods, [31] however results are not presented in the primary analysis due to small sample sizes and multicollinearity between sports-related significant predictors.

## Results

### Population and SARS-CoV-2 Positivity

During the investigation period, the school district identified 1,177 eligible contacts from grades K–12 (129 staff, 1,048 students) who were exposed to 90 school-associated index cases (Fig. 1). No in-school exposures were reported from the early learning center (pre-K). A total of 796 (68%) contacts completed the survey. Among these 796, 404 (51%) were male, 363 (46%) were Hispanic or Latino/a, 231 (29%) were non-Hispanic Black, and 178 (22%) were non-Hispanic White (Table 1). Ten percent of survey participants were staff and 90% were students (469 elementary, 121 middle, and 127 high school students); 483 (61%) participants were exposed to a student index case and 296 (37%) to a staff index case. All elementary school students and 94% of middle/high school students reported attending school in person, with 15 middle/high school students attending school only for athletics. Additional characteristics of participants are presented in Table 1.

**Table 1:** Demographics and characteristics of exposure among students and staff exposed in school to a COVID-19 case in a school district in Georgia, December 2020–January 2021 (N=796)

	Elementary Students *N*=469	Middle/High Students *N*=248	Staff *N*=79	Total *N*=796
**Demographics**				
Gender				
Male	239 (51.0%)	151 (60.9%)	14 (17.7%)	404 (50.7%)
Female	227 (48.4%)	97 (39.1%)	65 (82.3%)	389 (48.9%)
Missing	3 (0.6%)	-	-	3 (0.4%)
Race/ethnicity				
Non-Hispanic White	58 (12.4%)	71 (28.6%)	49 (62.0%)	178 (22.4%)
Non-Hispanic Black	127 (27.1%)	83 (33.5%)	21 (26.6%)	231 (29.0%)
Hispanic or Latino/a	268 (57.1%)	87 (35.1%)	8 (10.1%)	363 (45.6%)
Other category^a^	16 (3.4%)	7 (2.8%)	1 (1.3%)	24 (3.0%)
School level				
Elementary (grades K-5)	469 (100.0%)	-	52 (65.8%)	521 (65.5%)
Middle (grades 6-8)	-	121 (48.8%)	12 (15.2%)	133 (16.7%)
High (grades 9-12)	-	127 (51.2%)	10 (12.7%)	137 (17.2%)
District-level	-	-	5 (6.3%)	5 (0.6%)
**Characterstics of exposure**				
Index Case School Role				
Staff	220 (46.9%)	38 (15.3%)	38 (48.1%)	296 (37.2%)
Student	246 (52.5%)	202 (81.5%)	35 (44.3%)	483 (60.7%)
Missing	3 (0.6%)	8 (3.2%)	6 (7.6%)	17 (2.1%)
Reported primary exposure location^b^				
Classroom/educational^c^	369 (78.7%)	152 (61.3%)	50 (63.3%)	571 (71.7%)
Bus	98 (20.9%)	29 (11.7%)	1 (1.3%)	128 (16.1%)
Sports	1 (0.2%)	61 (24.6%)	4 (5.1%)	66 (8.3%)
Office/Meeting	-	-	23 (29.1%)	23 (2.9%)
Unknown	1 (0.2%)	6 (2.4%)	1 (1.2%)	8 (1.0%)
Concurrent exposure in school				
Exposed to one case in school at a time	442 (94.2%)	242 (97.6%)	78 (98.7%)	762 (95.7%)
Concurrently exposed to two cases in school	23 (4.9%)	4 (1.6%)	1 (1.3%)	28 (3.5%)
Concurrently exposed to ≥3 cases in school	4 (0.9%)	2 (0.8%)	0 (0.0%)	6 (0.8%)

^a^Includes participants who reported Asian or American Indian/Alaska Native identity, or unknown

^b^The school district assigned each contact a single predominant location for exposure

^c^Tutoring, after-school care, lunch

Detailed reasons for non-participation among the 381 contacts who did not complete the survey are included in Figure S1 in supplementary materials. Where data were available, the people who refused testing were similar to consenting participants by gender, staff vs. student role, and school level. Race/ethnicity differed between these populations; nearly half (49%) of people who refused were non-Hispanic White, 10% were non-Hispanic Black, and 36% were Hispanic or Latino/a compared to 22%, 29% and 46%, respectively, among participants.

Of the 796 surveyed participants, 628 (79%) completed COVID-19 testing (Fig. 1). Of these, 58 tested positive, for a positivity rate of 9.2%. Positivity was 5.9% (4/68) for staff, 9.0% (32/354) for elementary school students, and 10.7% (22/206) for middle/high school students (chi-squared P=0.5). Overall, 199 contacts refused COVID-19 testing (142 refused all participation, 57 refused testing only). The most frequent reason for refusal was the belief that testing was not necessary since the contact was asymptomatic (n=48, 24%). Detailed reasons for why participants refused are presented in Figure S1.

### Frequencies of Self-Reported Risk Behaviors in the 14 Days before In-School Exposure to a COVID-19 Case

Staff and students reported several in-school behaviors that could modify their exposure to SARS-CoV-2 or are known to be high risk for SARS-CoV-2 transmission. In the two weeks prior to exposure, 16% of the 796 participants reported participating in sports in school, including 3% (16/469) of elementary school students, 42% (103/248) of middle/high school students, and 11% (9/79) of staff (Table 2). The most frequent sports reported included indoor basketball and wrestling. Nearly half (49%) of participants reported unmasked time at school, including 24% who reported unmasked time indoors (not including meals); 13% reported unmasked time during school sports, which comprised 69% of elementary and 88% of middle/high school students who reported any sports participation. Among staff members, 65% reported indoor time with other staff, including 39% who had in-person meetings, 30% who ate lunch indoors, and 30% who reported other staff social time; 30% of staff reported unmasked time indoors with other staff, but all staff reported always wearing masks during in-person meetings.

**Table 2:** Frequencies of in-school and out-of-school risk behaviors among students and staff in the 14 days prior to in-school exposure to a COVID-19 case in a school district in Georgia, December 2020–January 2021 (*N*=796)

	Elementary Students *N*=469	Middle/High Students *N*=248	Staff *N*=79	Total *N*=796
**In-School Behaviors**				
Any sports participation^a^	16 (3.4%)	103 (41.5%)	9 (11.4%)	128 (16.1%)
Any non-sports extracurricular activities^b^	35 (7.5%)	21 (8.5%)	3 (3.8%)	59 (7.4%)
Ever took school bus to get to school	317 (67.7%)	132 (53.2%)	-	449 (56.5%)
Any unmasked time in school^c^	235 (50.5%)	109 (46.8%)	32 (43.8%)	376 (48.8%)
Any unmasked time in school indoors^c^	99 (21.3%)	74 (31.8%)	13 (18.1%)	186 (24.2%)
Any unmasked time in school outdoors	200 (51.7%)	62 (45.6%)	24 (61.5%)	286 (50.9%)
Any unmasked time in school sports	11 (2.4%)	91 (37.6%)	2 (2.6%)	104 (13.2%)
**Out of school extracurricular activities**				
Sports outside of school	15 (3.2%)	22 (8.9%)	1 (1.3%)	38 (4.8%)
Non-sports extracurriculars out of school	26 (5.6%)	33 (13.4%)	9 (11.4%)	68 (8.6%)
**History of travel**				
Traveled outside of state	13 (2.8%)	11 (4.5%)	9 (11.4%)	33 (4.2%)
**Visits to non-school indoor locations**				
Visited any indoor location	313 (66.9%)	177 (71.4%)	70 (89.7%)	560 (70.5%)
Grocery/retail	189 (40.4%)	99 (39.9%)	53 (67.1%)	341 (42.9%)
Restaurant	69 (14.7%)	60 (24.2%)	25 (31.7%)	154 (19.4%)
Church/religious locations	52 (11.1%)	28 (11.3%)	6 (7.6%)	86 (10.8%)
Gym	8 (1.7%)	22 (8.9%)	9 (11.4%)	39 (4.9%)
Club/bar	-	-	2 (2.5%)	2 (0.3%)
Other indoor location	41 (8.8%)	24 (9.7%)	8 (10.1%)	73 (9.2%)
Ever didn’t wear a mask in indoor locations	72 (15.4%)	70 (28.7%)	28 (35.4%)	170 (21.5%)
**Social gatherings with non-HH members**				
Any social gatherings with non-HH members	165 (35.2%)	108 (43.6%)	43 (54.4%)	316 (39.7%)
Any unmasked indoor social gatherings	149 (31.9%)	95 (38.8%)	40 (51.3%)	284 (36.0%)
Had social gatherings on ≥2 occasion	90 (19.2%)	62 (25.0%)	32 (40.5%)	184 (23.1%)
Had social gatherings on ≥5 occasions	31 (6.6%)	15 (6.1%)	5 (6.3%)	51 (6.4%)
Number of people in social gathering 1-2	47 (10.0%)	20 (8.1%)	9 (11.4%)	76 (9.6%)
Number of people in social gathering 3-5	51 (10.9%)	41 (16.5%)	15 (19.0%)	107 (13.5%)
Number of people in social gathering ≥6	66 (14.1%)	47 (19.0%)	19 (24.1%)	132 (16.6%)
**Staff-Specific Behaviors**				
Had in-person meetings with other staff^d^	-	-	30 (38.5%)	30 (38.5%)
Had lunch with other staff indoors	-	-	23 (29.9%)	23 (29.9%)
Spent time with other staff any other time	-	-	23 (29.9%)	23 (29.9%)
Spent any unmasked time with other staff	-	-	23 (29.9%)	23 (29.9%)

*HH* Household

*Individual numbers may not add to denominator if participants declined individual questions

^a^Reported sports include basketball, cheerleading, wrestling, other (both elementary and middle/high); martial arts (elementary only); baseball, track, football, lacrosse, soccer, swimming, tennis, volleyball (middle/high only)

^b^Extracurricular activities included after-school care, dance, band, choir, tutoring, drama club, theater

^c^Does not include meals

^d^Reported 100% mask use for in-school meetings with other staff

**Fig. 2:**
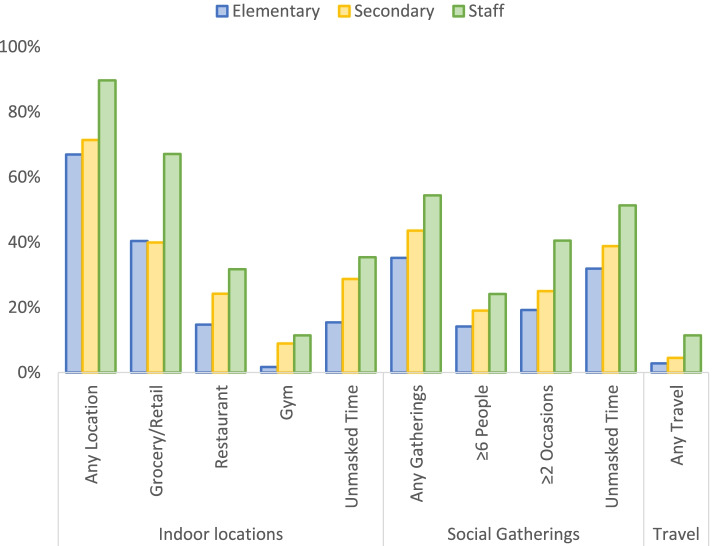
Proportions of students and staff reporting selected out-of-school risk behaviors in the 14 days prior to in-school exposure to a COVID-19 case in a Georgia school district, December 2020–January 2021 (N=796)

Staff and students also reported a range of out-of-school behaviors that have been identified as high risk for SARS-CoV-2 transmission. Four percent of participants reported travel outside of Georgia, including 11% of staff (Table 2, Fig. 2). Over 70% of participants reported visiting non-school indoor locations, including grocery/retail (43%), indoor restaurants (19%), church/religious locations (11%), and gyms (5%). These proportions increased by age group, with staff reporting the highest rates (90%) followed by middle/high school students (71%). Rates of unmasked time in indoor locations were 22% and also increased by age group. Forty percent of participants reported social visits with persons outside their household; 41% of staff reported ≥2 social visits and 24% attended gatherings of ≥6 people, versus 19-25% and 14-19% among students, respectively. Likewise, 51% of staff, 39% of middle/high, and 32% of elementary school students reported unmasked time indoors during social visits.

### Associations between SARS-CoV-2 Positivity, Self-Reported Behaviors, and Exposure Characteristics

**Fig. 3:**
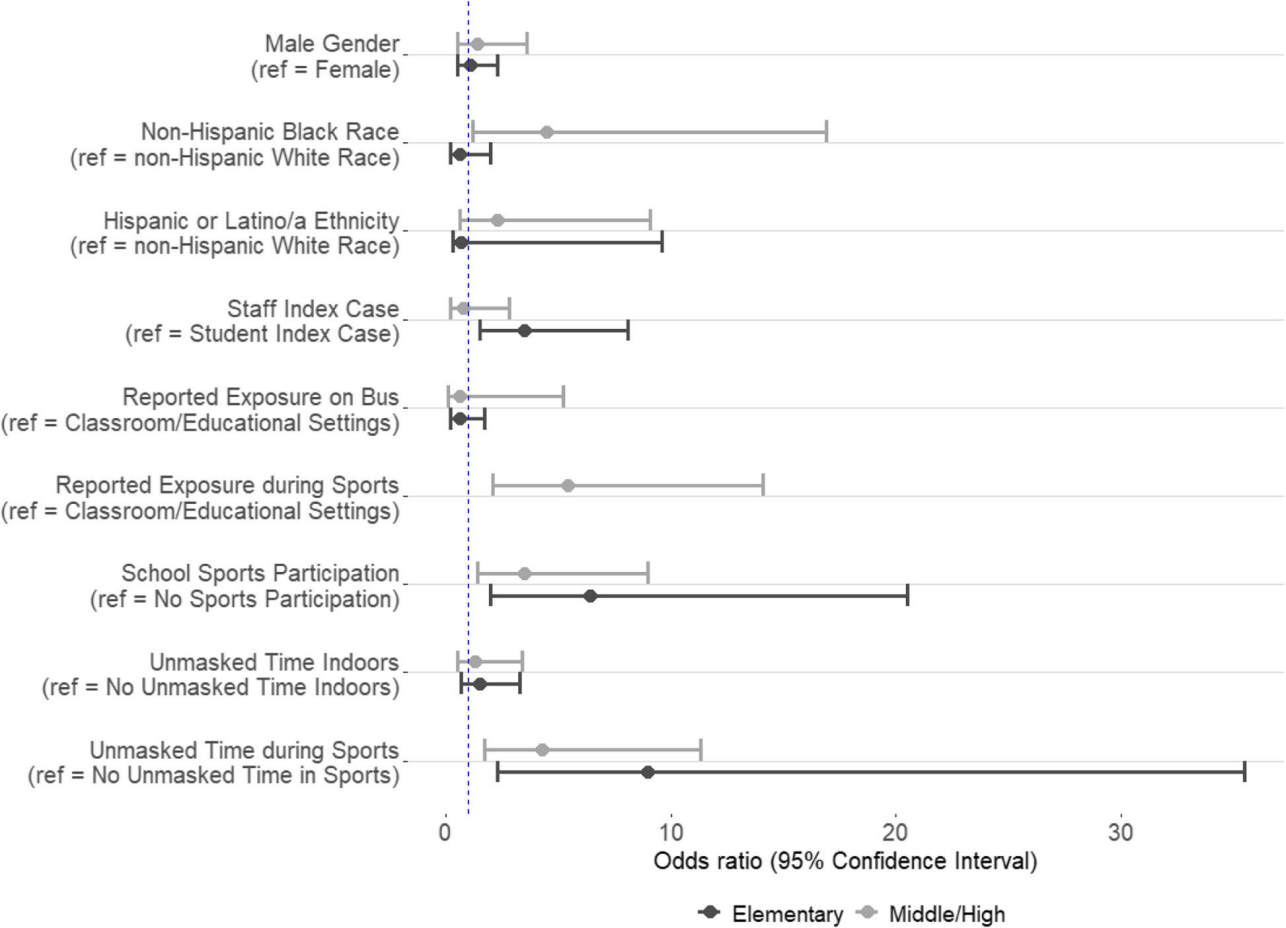
Associations between SARS-CoV-2 test positivity and participant demographics, school-reported characteristics of exposure, and selected in-school risk behaviors in the 14 days prior to in-school COVID-19 exposure among K–12 students in a school district in Georgia, December 2020–January 2021 (N=560). The dotted line represents an odds ratio of 1, and predictors with 95% confidence intervals that do not cross this line indicate statistical significance at the *P*=0.05 level

For elementary school students, positivity was 14% (24/172) among students exposed to a staff index case compared with 4% (8/182) if the index case was a student (odds ratio [OR=3.5], 95% confidence interval [CI]=1.5-8.1) (Table 3, Fig. 3). Positivity was 36% (5/14) among students who reported playing sports in school compared with 8% (27/339) among those who did not (OR=6.4, 95% CI=2.0-20.5), and positivity was 44% (4/9) among students who reported any unmasked time playing sports compared to 8% (28/344) among all other students (OR=9.0, 95% CI=2.3-35.5). When restricting this comparison to only the 14 tested students who reported playing sports, positivity remained 44% (4/9) among students who played sports without masks compared to 20% (1/5) among students who always wore a mask during sports.

**Table 3:** Associations between SARS-CoV-2 test positivity and demographics, characteristics of exposure, and behaviors in the 14 days prior to in-school exposure to a COVID-19 case among K–12 students in a school district in Georgia, December 2020–January 2021 (*N*=560)

	Elementary School Students				Middle/High School Students			
	(+) Contacts *N*=32	(-) Contacts *N*=322	OR (95% CI)	*P* value^	(+) Contacts *N*=22	(-) Contacts *N*=184	OR (95% CI)	*P* value^
**Demographics**								
**Gender**								
Male	17 (9.6%)	161 (90.4%)	1.1 (0.5-2.3)	0.7	15 (11.9%)	111 (88.1%)	1.4 (0.5-3.6)	0.5
Female	15 (8.5%)	161 (91.5%)	Ref		7 (8.8%)	73 (91.2%)	Ref	
**Race and ethnicity**								
Non-Hispanic White	6 (11.8%)	45 (88.2%)	Ref		3 (4.7%)	61 (95.3%)	Ref	
Non-Hispanic Black	7 (7.9%)	82 (92.1%)	0.6 (0.2-2.0)	0.4	12 (18.2%)	54 (81.8%)	4.5 (1.2-16.9)	***0.03***
Hispanic or Latino/a	17 (8.4%)	186 (91.6%)	0.7 (0.3-1.8)	0.5	7 (10.0%)	63 (90.0%)	2.3 (0.6-9.1)	0.3
Other race/ethnicity^a^	2 (18.2%)	9 (81.8%)	1.7 (0.3-9.6)	0.6	0 (0.0%)	6 (100.0%)	-	1.0
**Characterstics Of Exposure**								
**Index case role in school**								
Staff	24 (14.0%)	148 (86.0%)	3.5 (1.5-8.1)	***0.002***	3 (8.8%)	31 (91.2%)	0.8 (0.2-2.8)	1.0
Student	8 (4.4%)	174 (95.6%)	Ref		19 (11.1%)	153 (88.9%)	Ref	
**Reported primary exposure location**^b^								
Classroom/educational setting^c^	28 (9.8%)	258 (90.2%)	Ref		8 (6.3%)	120 (93.7%)	Ref	
Bus	4 (6.0%)	63 (94.0%)	0.6 (0.2-1.7)	0.5	1 (4.0%)	24 (96.0%)	0.6 (0.1-5.2)	1.0
Sports	0 (0.0%)	1 (100.0%)	-	1.0	13 (26.5%)	36 (73.5%)	5.4 (2.1-14.1)	***0.0002***
**Concurrent exposure in school**								
Concurrently exposed to ≥1 case in school	2 (10.5%)	17 (89.5%)	1.2 (0.3-5.4)	0.7	1 (25.0%)	3 (75.0%)	2.9 (0.3-28.9)	0.4
Exposed to 1 case in school	30 (9.0%)	305 (91.0%)	Ref		21 (10.4%)	181 (89.6%)	Ref	
**In-School Behaviors**								
**Transportation to/from school**								
Ever took bus	19 (8.2%)	214 (91.8%)	0.7 (0.4-1.5)	0.4	12 (10.8%)	99 (89.2%)	1.0 (0.4-2.5)	0.9
Never took bus	13 (10.7%)	108 (89.3%)	Ref		10 (10.5%)	85 (89.5%)	Ref	
**Participation in sports at school**								
Any sports participation	5 (35.7%)	9 (64.3%)	6.4 (2.0-20.5)	***0.0004***	15 (17.7%)	70 (82.3%)	3.5 (1.4-9.0)	***0.007***
No sports participation	27 (8.0%)	312 (92.0%)	Ref		7 (5.8%)	114 (94.2%)	Ref	
**Participation in non-sports extracurriculars**								
Any non-sports extracurricular activities^d^	3 (11.5%)	23 (88.5%)	1.3 (0.4-4.7)	0.7	1 (5.9%)	16 (94.1%)	0.5 (0.1-4.0)	1.0
No non-sports extracurricular activities	29 (8.9%)	298 (91.1%)	Ref		21 (11.1%)	168 (88.9%)	Ref	
**Mask use in school**								
Any unmasked time in school	20 (10.3%)	175 (89.7%)	1.4 (0.7-2.9)	0.4	10 (10.6%)	84 (89.4%)	1.1 (0.4-2.7)	0.9
No unmasked time in school	12 (7.6%)	146 (92.4%)	Ref		10 (9.9%)	91 (90.1%)	Ref	
Any unmasked time in school indoors	10 (11.8%)	75 (88.2%)	1.5 (0.7-3.3)	0.3	8 (11.9%)	59 (88.1%)	1.3 (0.5-3.4)	0.6
No unmasked time in school indoors	22 (8.2%)	246 (91.8%)	Ref		12 (9.4%)	116 (90.6%)	Ref	
Any unmasked time in school outdoors	17 (10.5%)	145 (89.5%)	1.5 (0.7-3.3)	0.3	3 (5.8%)	49 (94.2%)	0.4 (0.1-1.6)	0.3
No unmasked time in school outdoors	10 (7.4%)	126 (92.6%)	Ref		8 (12.9%)	54 (87.1%)	Ref	
**Mask use in school sports**								
Any unmasked time in school sports	4 (44.4%)	5 (55.6%)	9.0 (2.3-35.5)	***0.005***	15 (20.3%)	59 (79.7%)	4.3 (1.7-11.3)	***0.001***
No unmasked time in school sports^e^	28 (8.1%)	316 (91.9%)	Ref		7 (5.5%)	120 (94.5%)	Ref	

*OR* Odds ratio, *CI* Confidence interval

^chi-squared or Fisher Exact Test *P* value

^a^Includes participants who reported Asian or American Indian/Alaska Native identity, or unknown

^b^The school district assigned each contact a single predominant location for exposure

^c^Classroom, tutoring, after-school care, lunch

^d^Extracurricular activities included after-school care, dance, band, choir, tutoring, drama club, theater

^e^Denominator includes students who did not play sports and student who always wore a mask during sport

For middle/high school students, positivity was 27% (13/49) among students whose school-reported exposure setting was during sports compared to 6% (8/128) if reported exposure setting was the classroom (OR=5.4, 95% CI=2.1-14.1) or 4% (1/25) if reported exposure setting was on the school bus (OR=8.7, 95% CI=1.1-70.4) (Table 3, Fig. 3). The difference in positivity between exposure on the bus compared to the classroom was not statistically significant. Similarly, positivity was 18% (15/85) among students who reported playing sports in school compared to 6% (7/121) among students who did not (OR=3.5, 95% CI=1.4-9.0), and was 20% (15/74) among students who reported any unmasked time playing sports compared to 6% (7/127) among all other students (OR=4.3, 95% CI=1.7-11.3). When restricting this comparison to only the 85 tested students who reported playing sports, positivity remained 20% (15/74) among students who played sports without masks but was 0% (0/11) among students who always wore a mask during sports. Close-contact indoor sports were the most commonly reported among the 15 SARS-CoV-2 positive sports players, including basketball (53%, 8/15) and wrestling (33%, 5/15). Positivity was also higher among non-Hispanic Black students (18%) compared to non-Hispanic White students (5%) (OR=4.5, 95% CI=1.2-16.9); however, the proportion of Black students was three times higher among students who played sports (74%) than those who did not (26%), so this correlation is likely due to confounding and was not observed in multivariate analyses (Table S1).

There was no association for students of any age between SARS-CoV-2 positivity and gender, taking the school bus, participation in non-sports extracurriculars, general indoor mask use in school, or exposure to multiple cases in school (Table 3). Among elementary school students, there was also no association with race/ethnicity or reported exposure location, and there was no association with staff vs. student index case role for middle/high school students. Frequencies of risk behaviors by test result among staff and results of multivariate analyses are found in the appendices (Tables S1, S2).

## Discussion

This analysis builds on a previous investigation of in-school SARS-CoV-2 transmission to examine prevalence of risk behaviors among 717 students and 79 school staff originally identified as contacts of a COVID-19 case in a school setting. High rates of risk behaviors for school and community transmission were reported in this population despite high community incidence and lack of vaccine availability. A large proportion of participants reported unmasked time at school indoors, and most students who participated in sports did not wear masks during these activities. The majority of participants reported visiting indoor locations in the community, and large numbers reported social gatherings with people outside their household, including groups of ≥6 people.

This analysis also identified factors associated with SARS-CoV-2 positivity in this population of unvaccinated staff and students exposed to COVID-19 in school, including self-reported behaviors and school-reported characteristics of exposure. Nearly 10% of participants tested positive for SARS-CoV-2. Across student age groups, the strongest associations with SARS-CoV-2 positivity were with participation in school sports, especially unmasked time in sports, exposure to an index case during sports (middle/high only), and exposure to a staff index case (elementary only).

These results highlight behaviors that might increase or modify the risk of in-school transmission and introductions of SARS-CoV-2 into schools and help to demonstrate the connections between the school environment, in-school behaviors, and out-of-school behaviors. Since people who work in or attend schools are part of the community at-large, risk behaviors out of school could lead to increased opportunities for introductions in school and vice versa when community vaccination is low [8, 32, 33]. For instance, the high proportion of respondents who reported dining indoors at restaurants or having large social gatherings has implications for introductions in the school setting, particularly due to the high level of community transmission in winter of 2020-2021 and unvaccinated status of staff and students [27]. Correspondingly, although in-person education has not consistently been shown to increase community transmission, [8, 34–37] this investigation and others have found substantial transmission from positive contacts to their families [26, 27, 38, 39].

Few studies have examined behaviors in and out of school among a school population in the pre-vaccine era, which highlights the importance of these data in the literature. In October 2020, 65% of surveyed US middle and high school students reported consistent mask use in classrooms among their peers, and only 28% reported consistent mask use during sports or extracurricular activities [40]. In four studies from May-November 2020, 74-89% of surveyed US adults reported wearing a mask in public, 80-89% tried to keep 6 feet apart, 66-82% avoided restaurants, and 38% avoided socializing with people outside their household, with lower rates in rural areas [22–24, 41]. However, it is challenging to directly compare these studies to the current results due to varying study populations and the potential for changes in behavior patterns over the course of the pandemic.

One of the key results from this analysis was the strong association between participation in indoor sports and SARS-CoV-2 positivity. Indoor sports (basketball, wrestling) were the most frequently reported by participants, likely due to the winter sports season, and associations with SARS-CoV-2 positivity were very robust across age groups. Among middle/high school students, this result was consistent between self-reported sports behaviors and the exposure source identified by the school district. This was a surprising result for elementary school students, who do not have formal interscholastic sports leagues and were not generally identified as having predominantly sports-related exposures by the school district; however, the association with self-reported sports participation was very strong despite small sample sizes. This could be explained because, unlike in middle and high school, students in elementary school stay together in a cohort throughout the day including activities like recess, so the school district may have identified the classroom as the predominant exposure location due to the long duration of time in this setting. However, this designation does not preclude additional exposures during other activities. Thereby, although it is not known whether participants played sports directly with the index case, the strong associations between SARS-CoV-2 positivity and self-reported sports participation among this population of children with a known exposure suggests that playing sports unmasked conferred an additional risk to sitting in the classroom.

These findings of potential increased risk associated with sports are consistent with previous reports, which provide a growing body of evidence that there is limited ability to prevent transmission in unvaccinated individuals during high-intensity, close-contact indoor sports [12, 27, 32, 42–44]. When community transmission is high, other athletic activities could be considered where comprehensive multilayered prevention strategies can be implemented, including correct and consistent mask use, vaccination of eligible staff and students, adequate physical distancing, avoidance of large crowds, and improved ventilation [7]. Students can thereby continue to experience the physical and mental health benefits of school athletic activities while mitigating the risk to themselves and others [45]. Mask use during sports did appear to be protective in this unvaccinated population, an important finding that could be incorporated into future prevention practices; however, results should be verified with larger studies.

These results also support previous findings that school staff are central to transmission in elementary schools [8, 26, 27, 46, 47]. Close interactions between teachers and younger students are necessary for learning but provide more opportunities for transmission among unvaccinated staff or students, particularly if mask use is not consistent. Despite school policies requiring mask use indoors, our findings and others indicate that these policies may not be followed with 100% fidelity, particularly among younger students [14, 40]. This issue is compounded by the finding that risk behaviors in the community were highest among staff. Interventions to reduce risks of staff-related transmission include vaccination of eligible staff and students, activities to reinforce appropriate mask use, reducing unnecessary in-person interaction among unvaccinated staff, and taking measures to reduce community exposures.

As a final note, the high prevalence of risk behaviors identified in this investigation underscore the importance of comprehensive school, state, and local policies to reduce transmission, in keeping with guidelines to prioritize schools remaining open for in-person instruction over nonessential activities [7]. At the time of the investigation, vaccination was not available to staff or students, and Georgia COVID-19 regulations did not include any universal mask mandate or prohibit dining indoors in restaurants or the operation of indoor gyms and bars, [48] despite demonstrated efficacy in reducing community transmission [15–19]. The frequency of these behaviors in our population are therefore not unexpected given the proximity to holidays, cold winter weather, and ongoing effects of isolation, particularly among staff who may live alone or have responsibilities outside of the home [49, 50]. Similarly, although CDC recommends limiting sports and extracurricular activities when community transmission is high, [7] the Georgia High School Association did not impose restrictions on school sports or require mask use during sports at the time of the investigation [51]. Without this guidance, it may have been challenging for local school boards to independently limit sports or require mask use during athletics. Implementing structural policies at the state or local level during periods of high transmission would likely improve adherence to behavioral recommendations, reduce community acquisition of SARS-CoV-2, and therefore reduce introductions of COVID-19 into schools [16, 52]. Improving vaccination rates among eligible populations may also reduce introductions into schools and in-school transmission. However, until vaccination is available to persons of all ages, continued adherence to in-school prevention measures such as appropriate mask use will continue to be important to prevent in-school transmission.

This investigation had several limitations. Enrollment was limited to known contacts of a positive SARS-CoV-2 case and occurred in a single school district, which constrains generalizability to other populations. Similarly, the investigation occurred prior to widespread vaccine availability, and both behaviors and risk factors have likely shifted since this time. The sample size of SARS-CoV-2 positive participants was also small, which limited the ability to conduct statistical comparisons, and the use of self-reported survey data rather than direct observation of risk behaviors increases the chance of recall bias and social desirability bias. Due to the sampling design of recruiting only people exposed in school, it was not feasible to assess associations between SARS-CoV-2 positivity and out-of-school behaviors due to the risk of selection bias. Furthermore, for all self-reported in-school behaviors, it is not known whether the participant was exposed to the positive case during those activities, so this investigation could only identify associations but cannot determine causality. A sizeable percent of the population also could not be reached or declined participation, with the leading reason for refusals being belief that testing was not needed for asymptomatic contacts. This may indicate that contacts who refused were different than participants regarding behaviors for SARS-CoV-2 prevention. Finally, although SARS-CoV-2 positivity was associated with sports participation, comparisons between individual sports activities or settings could not be conducted due to low sample size and the limited number of sports in season during the investigation. Future studies could attempt to discern which sports activities are associated with the highest risk for transmission.

Despite these limitations, this investigation is one of the more comprehensive reports of school staff and student behaviors relevant to COVID-19 in the literature to date and identifies several characteristics and behaviors associated with probable SARS-CoV-2 transmission in school settings. These findings may be valuable to guide implementation of interventions in and out of schools to improve the safety of staff and student populations. Furthermore, COVID-19 vaccination is still not available for children under 5 years, and vaccination rates remain low in many US counties and internationally. Additional studies are needed to assess the impact of behaviors in school and the community on transmission in school populations, including the role of vaccination status.

## Conclusions

The results of this investigation underscore the importance of both in-school prevention measures and individual behaviors for COVID-19 in school settings. In a population of staff and students with an in-school exposure, risk behaviors were common despite high community transmission, and SARS-CoV-2 positivity was associated with several of these behaviors including participation in sports. It is possible that these behaviors may thereby contribute to in-school transmission or introductions of SARS-CoV-2 into the school. To mitigate these risks and maintain the safety of in-person learning, comprehensive school and community prevention measures are needed. These include vaccination of eligible staff and students and measures to reduce transmission in unvaccinated populations including correct and consistent mask use, reduction of high-risk activities such as indoor school sports, and limiting social gatherings outside of school. Future research is also needed to continue to examine the impact of risk behaviors on school transmission where vaccination is widely available.

## Supplementary Information

**Additional file 1.**

## Data Availability

The datasets generated and/or analyzed during the current study are not publicly available to protect the confidentiality of study participants including minor children but are available from the corresponding author upon reasonable request.

## Abbreviations

CDC: Centers for Disease Control and Prevention
OR: Odds ratio
CI: Confidence Intervals
