# Supplementary material for: Prevalence of risk behaviors and correlates of SARS-CoV-2 positivity among in-school contacts of confirmed cases in a Georgia school district in the pre-vaccine era, December 2020–January 2021
Source: BMC Public Health. 2022 Jan 14;22:101. doi: 10.1186/s12889-021-12347-7 (PMC8759220; doi:10.1186/s12889-021-12347-7)
Supplement: Supplementary file 1 — Additional file 1. [file 12889_2021_12347_MOESM1_ESM.docx]

Additional File

Figure S1: Detailed reasons for refusal of COVID-19 testing and/or survey participation among school staff and students in a school district in Georgia, December 2020–January 2021. Categories are mutually exclusive.

**Refused testing and/or all participation (n=199)**

- Doesn’t want testing since child asymptomatic (n=48, 24.1%)
- Was tested/wants testing elsewhere (n=33, 16.6%)
- Not available/too busy (n=15, 7.5%)
- Quarantine almost over/testing won’t allow return to school earlier (n=8, 4.0%)
- Previous contact, doesn’t want repeat participation (n=5, 2.5%)
- Child doesn’t want test/afraid of pain (n=4, 2.0%)
- Doesn’t think child really exposed (n=4, 2.0%)
- Doesn’t think testing necessary (n=4, 2.0%)
- Doesn’t believe/trust test results (n=3, 1.5%)
- Doesn’t want to know results (n=2, 1.0%)
- Already had COVID-19 (n=2, 1.0%)
- Doesn’t believe in COVID-19 (n=1, 0.5%)
- Had symptoms and so doesn’t need test (n=1, 0.5%)
- No transportation (n=1, 0.5%)
- Unknown/no reason given (n=67, 33.7%)

**Refused only survey, completed testing (n=42)**

- Repeat participant, didn’t want to repeat survey (n=17, 40.4%)
- Unknown/no reason given (n=25, 59.6%)

Table S1: Multivariate logistic regression models among K–12 students exposed to COVID-19 in a school district in Georgia, December 2020–January 2021 (N=554, real-time polymerase chain reaction [RT-PCR] positive = 54, RT-PCR negative = 500). Multiple model results are presented due to multicollinearity among predictors relating to participation in sports and/or exposure during sports in school. Models are presented in descending order of Akaike information criterion (AIC) optimization with Model A having the best AIC optimization.

|  | **aOR** | **95% CI** | **P value** |
| --- | --- | --- | --- |
| **Model A** |  |  |  |
| Staff Index Role | 2.5 | 1.4, 4.6 | 0.003 |
| Male gender | 1.0 | 0.6, 1.9 | 0.9 |
| Non-Hispanic Black^1^ | 1.9 | 0.8, 4.6 | 0.6 |
| Hispanic or Latino/a^1^ | 2.0 | 0.8, 4.9 | 0.5 |
| Other race/ethnicity^1^ | 1.8 | 0.3, 9.6 | 0.9 |
| Any unmasked sports in school | 5.4 | 2.6, 11.5 | <0.001 |
| **Model B** |  |  |  |
| Staff Index Role | 2.6 | 1.4, 4.8 | 0.002 |
| Male gender | 1.0 | 0.6, 1.9 | 0.9 |
| Non-Hispanic Black^1^ | 2.0 | 0.8, 4.7 | 0.5 |
| Hispanic or Latino/a^1^ | 1.9 | 0.8, 4.6 | 0.5 |
| Other race/ethnicity^1^ | 1.8 | 0.3, 9.5 | 0.9 |
| Any sports in school | 4.6 | 2.3, 9.5 | <0.001 |
| **Model C** |  |  |  |
| Staff Index Role | 2.3 | 1.2, 4.4 | 0.001 |
| Male gender | 0.9 | 0.5, 1.7 | 0.8 |
| Non-Hispanic Black^1^ | 1.5 | 0.6, 3.6 | 0.6 |
| Hispanic or Latino/a^1^ | 1.5 | 0.6, 3.3 | 0.7 |
| Other race/ethnicity^1^ | 1.4 | 0.3, 7.3 | 0.9 |
| Exposure during school sports^2^ | 5.3 | 2.2, 12.6 | 0.002 |
| Exposure on school bus^2^ | 0.8 | 0.3, 2.2 | 0.06 |

aOC=adjusted odds ratio; ^1^ Compared to non-Hispanic White; ^2^ compared to classroom/educational setting exposure

Table S2: Frequencies of SARS-CoV-2 test positivity and negativity among school staff by demographics, risk factors, and in-school behaviors during the 14 days before exposure among participants in an investigation on in-school transmission in a school district in Georgia, December 2020–January 2021 (N=68). No statistical comparisons are conducted due to low sample size.

|  | **Staff** | |
| --- | --- | --- |
|  | **(+) Contacts N=4** | **(-) Contacts N=64** |
| **Gender^1^** |  |  |
| Female | 3 (5.3%) | 54 (94.7%) |
| Male | 1 (9.1%) | 10 (90.9%) |
| **Race and ethnicity** |  |  |
| Non-Hispanic White | 1 (2.4%) | 40 (97.6%) |
| Non-Hispanic Black | 0 (0.0%) | 19 (100.0%) |
| Hispanic or Latino/a | 3 (37.5%) | 5 (62.5%) |
| **Grade level** |  |  |
| Elementary | 3 (7.1%) | 39 (92.9%) |
| Middle | 0 (0.0%) | 11 (100.0%) |
| High | 1 (10.0%) | 9 (90.0%) |
| School district-level | 0 (0.0%) | 5 (100.0%) |
| **Index role in school** |  |  |
| Staff | 4 (11.8%) | 30 (88.2%) |
| Student | 0 (0.0%) | 34 (100.0%) |
| **Exposure location** |  |  |
| Classroom/Educational setting^2^ | 0 (0.0%) | 40 (100.0%) |
| Bus | 0 (0.0%) | 1 (100.0%) |
| Sports | 0 (0.0%) | 4 (100.0%) |
| Office/Meeting | 4 (17.4%) | 19 (82.6%) |
| **Participation in non-sports extracurriculars** |  |  |
| Any non-sports extracurricular activities | 0 (0.0%) | 2 (100%) |
| No non-sports extracurricular activities | 4 (6.1%) | 62 (93.9%) |
| **Participation in sports at school** |  |  |
| Any sports participation | 0 (0.0%) | 8 (100%) |
| No sports participation | 4 (6.7%) | 56 (93.3%) |
| **Mask use in school** |  |  |
| Any unmasked time in school | 3 (12.0%) | 22 (88.0%) |
| No unmasked time in school | 1 (2.6%) | 37 (97.4%) |
| Any unmasked time in school indoors | 1 (8.3%) | 11 (91.7%) |
| No unmasked time in school indoors | 3 (6.0%) | 47 (94.0%) |
| Any unmasked time in school outdoors | 3 (16.7%) | 15 (83.3%) |
| No unmasked time in school outdoors | 0 (0.0%) | 12 (100.0%) |
| **Mask use in school sports** |  |  |
| Any unmasked time in school sports | 0 (0.0%) | 2 (100%) |
| No unmasked time in school sports | 4 (6.1%) | 61 (93.9%) |

No statistical comparisons conducted due to low sample size; ^1^ Reported as self-identified gender, biological sex was used if gender was not reported; ^2^ classroom, tutoring, after-school care, lunch

**Assessing School-Based Transmission:**

**Contact Investigation Questionnaire**

Interviewer name:

| **CDC Internal Tracking ID (PTID):**  Contacts primary language: __________________ |
| --- |

***What is your Ethnicity:***

Hispanic or Latino

Not Hispanic or Latino

***What is your Race (select all that apply):***

American Indian or Alaska Native

Asian

Black or African American

Native Hawaiian or Other Pacific Islander

White

Other

I don’t know

***Gender Identity***

**What sex were you assigned at birth, on your original birth certificate?**

Male

Female

Refused

I don’t know

**Do you currently describe yourself as male, female, or transgender?**

Male

Female

Transgender

None of these

**Just to confirm, you were assigned {FILL} at birth and now you describe yourself as {FILL}, is that correct?**

Yes

No

Refused

I don’t know

***Role at school:***

Student

Teacher

Janitorial staff

School Nurse

Administration staff

Bus driver

Athletic Coach

Other: _____ specify

***Specify grade level if student/teacher:***

Kindergarten 1 2 3 4 5 6 7 8 9 10 11 12

Special ed; (specify grade level if Special Ed: ___________)

Other (specify: ____________)

**Which of these learning models have you participated in in the last 14 days: [14 days before sent home]**

Fully in person

Hybrid

Fully remote

Other (specify):__________

**Do you mostly stay with the same class during school day or do you take classes with different groups of students?**

Same group of students

Switch between different groups of students

Unknown

**What was the last date you attended school?**

**Date: _____**

**Do you attend classes/after school programs or work at any other schools?**

Yes

No

Unknown

**How do you get to school?**

School bus

Walk

Carpool with other school children, school staff, or teachers

Car alone or with only household members

Other (Specify: _______)

**Are you involved in any extra curricular activities at school?** Yes No

After-school sports at school (if yes, specify which sports: _____________________)

Choir

Band

Dance

After-school club (e.g. Chess, Debate)

Other (specify: ____________)

**Are you involved in any regular activities outside of school (eg: sports team, karate class, job, other clubs/group activities?**

Yes

No

*If yes:*

Sports (specify: ____)

Job

Choir

Band

Other group activities: (specify: ___-_)

[Say: *The next questions are about times you have spent with people outside of your household in last 14 days]*

**In the last 14 days, outside of school, how many times have you spent time with a friend/family member or someone else you don’t live with; this could include having family and friends over to your house or spending time at other people’s houses: ___**

*If >0 times:*

**Of these times, what was the largest group of people outside your household you were with?**

1-2

3-5

6-10

>10

**How much of the time were you wearing a mask when you were with people who do not live with you?**

All the time

Almost all of the time

Some of the time

Almost none of the time

**How much of the time were others wearing a mask?**

All the time

Almost All of the time

Some of the time

Almost none of the time

**How much of the time did you spend outdoors as opposed to indoors during these visits?**

All outdoors

Some indoor, some outdoors

All indoors

[Say: *The next questions* are *about your activities in the last 14 days*]_

**Have you been inside a store shopping for items (groceries, prescriptions, home goods, clothing, etc.)**

Yes

No

Don’t remember

*If yes:*

**Did you use a mask when you were in this location?**

All the time Almost all of the time some of the time Almost none of the time

**About how many people were wearing a mask?**

Almost none About half Almost all All

**About how many were staying six feet apart?**

Almost none About half Almost all All

**Have you been to church or a religious gathering/place of worship (church)?**

Yes

No

Don’t remember

*If yes:*

***Did you use a mask when you were in this location?***

All the time Almost all of the time some of the time Almost none of the time

**About how many people were wearing a mask?**

Almost none About half Almost all All

**About how many were staying six feet apart?**

Almost none About half Almost all All

**Have you been to a restaurant other than a drive through?**

Yes – Indoor

Yes- Outdoor

No

Don’t remember

*If yes:*

**Did you use a mask when you were in this location?**

All the time Almost all of the time some of the time Almost none of the time

**About how many people were wearing a mask?**

Almost none About half Almost all All

**About how many were staying six feet apart?**

Almost none About half Almost all All

**Have you been to a coffee shop other than a drive through?**

Yes – Indoor

Yes-Outdoor

No

Don’t remember

*If yes:*

**Did you use a mask when you were in this location?**

All the time Almost all of the time some of the time Almost none of the time

**About how many people were wearing a mask?**

Almost none About half Almost all All

**About how many were staying six feet apart?**

Almost none About half Almost all All

**Have you been to a bar or club?**

Yes – Indoor

Yes- Outdoor

No

Don’t remember

*If yes:*

**Did you use a mask when you were in this location?**

All the time Almost all of the time some of the time Almost none of the time

**About how many people were wearing a mask?**

Almost none About half Almost all All

**About how many were staying six feet apart?**

Almost none About half Almost all All

**Have you been to a gym or fitness center?**

Yes

No

Don’t remember

**Did you use a mask when you were in this location?**

All the time Almost all of the time some of the time Almost none of the time

**About how many people were wearing a mask?**

Almost none About half Almost all All

**About how many were staying six feet apart?**

Almost none About half Almost all All

**Have you had any international or domestic travel outside of your state in the last two weeks:**

Yes No (If yes, details on location of travel)_______

**Before you were identified as a contact of a COVID-19 case *(check all that apply)*:**

Did you wear face masks at school: Always Almost always Sometimes Almost never N/A

During bus ride to and from school : Always Almost always Sometimes Almost never N/A

During carpool ride to and from school: Always Almost always Sometimes Almost never N/A

While in class: Always Almost always Sometimes Almost never N/A

During school outdoor activities such as recess or gym class: Always Almost always Sometimes Almost never N/A

During singing or choir at school: Always Almost always Sometimes Almost Never N/A

During any before or afterschool care or activities: Always Almost Always Sometimes Almost Never N/A

During sports team participation: Always Almost always Sometimes Almost never N/A

During other extra-curricular activities at school that I have not mentioned: Always Almost always Sometimes Almost never N/A

**Are there times when you do not wear your mask at school?**

Yes

No

*If yes,*

What are those times? _____________________________

*If teacher/staff:*

**Did you attend in-person meetings with other staff?**

Yes

No

*--if yes--*

**Did you wear a mask when you were in-person meetings other staff?**

Always

Almost Always

Sometimes

Almost Never

**Do you eat lunch with other staff/spend time in a break room together**?

Yes

No

*If yes—*

**Did you wear a mask when you were in this situation?**

Always

Almost Always

Sometimes

Almost Never

**Are there any other times where you were around other staff?**

**Yes**

**No**

*If yes --*

**Did you wear a mask when you were in this situation?**

Always

Almost Always

Sometimes

Almost Never

**Do you currently have any symptoms of COVID-19? Symptoms include Fever, chills, cough, shortness of breath, difficulty breathing, fatigues, muscle aches, rigors, headaches, new loss of smell, new loss of taste, sore throat, congestion/runny nose, nausea, vomiting, diarrhea, and abdominal pain.**

Yes

No

Refused

Unknown

If yes: **Symptoms consistent with COVID-19 (check all that apply)**

Measured fever (defined as ≥100.4 oF)

Subjective fever (felt feverish)

Chills

Cough

Shortness of breath

Difficulty breathing

Fatigue

Muscle or body aches

Rigors

Headaches

New loss of taste

New loss of smell

Sore throat

Congestion/runny nose

Nausea/vomiting

Diarrhea

Abdominal pain

Other symptoms

Date of symptom onset__________

Do you have any Pre-existing medical conditions

Yes No *(If yes…)*

premature birth

lung disease (including asthma)

diabetes mellitus

cardiovascular disease (heart conditions)

kidney disease

liver disease

immunocompromising conditions

cancer

immunosuppressive therapy/medications

neurologic/neurodevelopmental disorder

other chronic diseases ____________

any other medical conditions as a child_________

**Have you ever been diagnosed with COVID-19?** Yes No

*If yes****,* date of diagnosis: ______**

**If yes,**

**Did you require any of the following for your care related to COVID-19 (check all that apply)**

Primary care telemedicine visit

Primary care office visit

ER visit

Hospitalization >24h [start/end dates___________]

ICU

Intubation

None of the above

**Have you been tested for COVID 19 in the last 14 days? Yes NO**

**If Yes, date test was taken**

**If yes, what was the result?**

Positive

Negative

Indeterminate

Unknown

**Household setting:**

Number of adults in the household ____

Number of children under 18 in the household _____

Number of children attending in person school or daycare: ________

Have any household contacts tested positive for COVID-19 in the last 14 days:

Yes number________ No

Have any non-household contacts test positive for COVID-19 in the last 14 days (besides school contact):

Yes number________ No

*If yes,*

Who tested positive (check all that apply):

|  | Date of positive result | Notes |
| --- | --- | --- |
| Parent/Guardian 1 |  |  |
| Parent/Guardian 2 |  |  |
| Sibling |  | Specify age: ______________ |
| Sibling |  | Specify age: ______________ |
| Sibling |  | Specify age: ______________ |
| Spouse/partner |  |  |
| Child |  | Specify age: ______________ |
| Child |  | Specify age: ______________ |
| Child |  | Specify age: ______________ |
| Other household contact |  | Specify other: _____________ |
| Other household contact |  | Specify other: _____________ |
| Non-household contact |  | Specify other: _____________ |
| Non-household contact |  | Specify other: _____________ |
